# Development and validation of a Delphi consensus-based questionnaire for the multidisciplinary management of type 2 inflammation-related diseases

**DOI:** 10.3389/falgy.2025.1543504

**Published:** 2025-04-03

**Authors:** Francisco Javier Ortiz de Frutos, Carolina Cisneros, José Miguel Villacampa, Óscar Palomares, Ignacio Dávila

**Affiliations:** ^1^ Dermatology Department, University Hospital 12 de Octubre, Madrid, Spain; ^2^ Pneumology Department, La Princesa Hospital, Madrid, Spain; ^3^ ENT Department, Fundación Jiménez Díaz University Hospital, Madrid, Spain; ^4^ Department of Biochemistry and Molecular Biology, School of Chemistry, Complutense University of Madrid, Madrid, Spain; ^5^ Allergy Service, University Hospital of Salamanca, Red de Enfermedades Inflamatorias, ISCIII, Departamento de Ciencias Biomédicas y del Diagnóstico, Instituto de Investigación Biosanitaria de Salamanca, Salamanca, Spain

**Keywords:** T2 inflammation, management, multidisciplinary, screening, questionnaire

## Abstract

**Objective:**

This study aimed to validate a 15-item screening questionnaire for the early detection of coexisting type 2 (T2) inflammatory diseases, such as asthma, atopic dermatitis, and chronic rhinosinusitis with nasal polyps (CRSwNP), among others.

**Methods:**

The questionnaire, designed through expert consensus by a scientific committee, underwent Delphi methodology for validation. A multidisciplinary panel of 19 clinicians from different specialties reviewed the questionnaire for clinical relevance, while 39 patients from different regions of Spain evaluated its comprehensibility.

**Results:**

The clinician panel reached a consensus on the relevance of 13 out of 15 items in the first round and agreed that a single positive response was sufficient to justify referral to the appropriate specialist. Two items were modified and validated in the second round. The patient panel unanimously agreed on the comprehensibility of the questionnaire in the first round. Linguistic variations were also ranked to ensure clarity across regions, further enhancing the validation of the tool.

**Conclusion:**

This validated questionnaire offers a practical tool for early detection of T2 inflammatory diseases. Its simplicity and comprehensibility, confirmed by clinicians and patients, make it suitable for use in various healthcare settings, supporting timely specialist referrals and improved patient care. Future studies will evaluate its effectiveness in real-world clinical practice.

## 1 Introduction

Dysregulation of type 2 (T2) immune responses, a natural defense mechanism under normal circumstances, can lead to pathological inflammation and is implicated in the pathophysiology of various diseases, such as asthma, atopic dermatitis (AD), and chronic rhinosinusitis with nasal polyps (CRSwNP), among others (1, 2). This common endotype partly explains the frequent coexistence and similar pathophysiological features of these entities. Despite advances in understanding the mechanisms driving T2 inflammation, leading to the development of targeted therapeutic strategies, significant unmet needs persist (3). In this respect, inadequate interdisciplinary communication and the absence of effective detection tools can delay diagnosis, resulting in suboptimal patient care. In fact, patients themselves have emphasized the necessity for interdisciplinary collaboration in managing T2-mediated diseases (4).

In response to this need, we recently developed a short questionnaire based on the cardinal symptoms of T2-mediated inflammatory diseases, in order to promote the early detection of coexisting T2 pathologies (5). Briefly, a comprehensive literature review was performed and expert meetings were conducted to develop a 15-item questionnaire to identify the eight most prevalent T2 diseases: asthma, CRSwNP, allergic rhinitis, allergic conjunctivitis, IgE-mediated food allergy, AD, eosinophilic esophagitis (EoE), and nonsteroidal anti-inflammatory drug-exacerbated respiratory disease (N-ERD). The questionnaire was drafted using patient-friendly language that allows them to identify and describe their symptoms easily. This tool, designed for clinical practice in Spain, is intended to be implemented across all levels of care, from primary to specialized settings, in both public and private healthcare systems, to support the initial screening of patients for suspected coexisting T2 diseases.

The present study aims to validate this questionnaire's item construction and language choice.

## 2 Methods

### 2.1 Study design and panel formation

**Figure 1:**
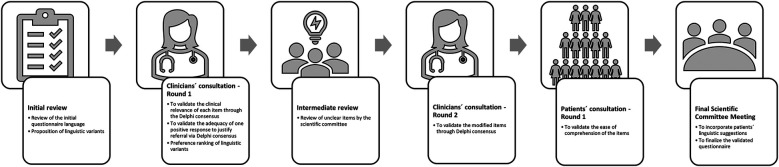
Workflow of the validation process.

A schematic workflow of the study is shown in Figure 1. First, the language and construction of items from the initial questionnaire were reviewed by the scientific committee previously responsible for its development (5) to identify synonyms and alternative phrasings for each concept. Subsequently, the clinical relevance of the selected items and the proposal that one positive answer to any of the questionnaire items would be sufficient for specialist derivation were tested in a Delphi consultation conducted with a panel of physicians from each specialty. The previously identified linguistic variants were also assessed through a simple preference ranking. After the clinicians validated the questionnaire, a selection of patients from patient advocacy groups were invited to validate its ease of comprehension, also through a Delphi consultation. These consultation rounds were conducted through a survey implemented on the SurveyMonkey platform (https://www.surveymonkey.com/).

The scientific committee members recommended three to seven experts from each relevant specialty to form the panel of physicians, dispersed geographically across the entire national Spanish territory. Those who accepted the invitation to participate formed a multidisciplinary panel of 19 clinicians. The panel included four otolaryngologists, three dermatologists, four pulmonologists, six allergists, and two immunologists. A modified questionnaire was developed on the basis of on the survey results from this panel. The revised questionnaire underwent a Delphi consultation process with a panel of 39 patients from patient associations diagnosed with at least one T2-related pathology. Five associations were contacted by phone to explain the study and invite participation. Subsequently, an email was sent with further details, requesting that each one selected at least five patients (adults with moderate to severe conditions). The Delphi methodology was outlined, highlighting the need for committed participants due to the potential for two questionnaire rounds. A follow-up email containing the link to the questionnaire was then provided. The associations were tasked with overseeing the selected participants, ensuring that only those who completed the first round would be eligible to respond to a second round, if necessary.

### 2.2 Delphi process and consensus criteria

The Delphi process involved iterative rounds of feedback and consensus from the multidisciplinary panels of experts and patients to ensure the questionnaire's relevance, clarity, and practical utility and to validate and refine the tool effectively. Based on the “BIOMED Concerted Action on Appropriateness” definitions for different panel sizes, the criteria for disagreement and agreement were established for a panel size of 19 and 39 clinicians and patients, respectively (6).

The Rand/UCLA appropriateness method was used for consensus analysis within the panels (6). Each item of the questionnaire was classified as “Appropriate”, “Uncertain”, or “Inappropriate”, based on the median score of the panel and the degree of agreement among panelists. Thus, items were categorized as “Appropriate”, if the median score ranged from 7–9, “Uncertain”, if the median score ranged from 4–6 or any median in disagreement, and “Inappropriate”, if the median score ranged from 1–3. The criteria for the degree of agreement were as follows: “Disagreement” if the median fell at one extreme (ranges 1–3 or 7–9) and the number of ratings at the opposite extreme was greater than or equal to one-third of the panel size or if the median fell in the range 4–6 and the number of ratings at one of the extremes was greater than or equal to one-third of the panel size. “Agreement” was established when the number of ratings outside the range containing the median was less than one-third of the panel size. A “Neutral” classification was used when neither agreement nor disagreement was achieved.

### 2.3 Evaluation metrics and data analysis

The clinicians' evaluation encompassed several aspects. Sociodemographic variables, including age, gender, region, hospital level, specialty, and years of experience were recorded. Clinicians then assessed the relevance of questionnaire items and if one positive response was sufficient for referral. Panelists also provided a ranking of linguistic variants of technical terms to ensure clarity for patient understanding.

Patients evaluated the comprehensibility of the questionnaire from their perspective and provided sociodemographic and clinical data. Patients were also given the opportunity to provide comments on each item of the questionnaire, so that they could express any doubts they had about the questions or offer suggestions regarding the clarity and ease of understanding.

## 3 Results

The panel of clinicians consisted of 19 specialists whose sociodemographic characteristics are shown in Table 1. In the first Delphi round, consensus was reached on two aspects: the clinical relevance of each questionnaire item (*n* = 15) for referral to another specialist and the sufficiency of a single positive response to justify referral. Two questionnaire items failed to reach a consensus regarding their clinical relevance and were returned to the scientific committee for modification. Item number 1 was deemed “uncertain” (most of the panel doubted the appropriateness of the statement's wording) and reached “disagreement” (most of the panel was unsure about the relevance of this item for referral to the corresponding specialist). Item number 13 was considered “appropriate” in terms of the statement's correctness but “neutral” (there was no agreement within the panel regarding the relevance of this item for referral). These two items were then modified and resubmitted to the clinician's panel in Delphi round 2. They were then considered appropriate and achieved consensus. Additionally, different linguistic options were explored for every item, and the most preferred option for each item was incorporated into the new version of the questionnaire (Supplementary Table S3).

**Table 1:** Demographic characteristics of the panel of clinicians.

Characteristics	Total (*N* = 19)
Age	
35–44 years	4 (21.1%)
45–54 years	5 (26.3%)
55–64 years	9 (47.4%)
Over 65 years	1 (5.3%)
Gender	
Male	9 (47.4%)
Female	10 (52.6%)
Region of practice	
Andalusia	5 (26.3%)
Canary Islands	4 (21.1%)
Cantabria	2 (10.5%)
Catalonia	1 (5.3%)
Valencia Community	4 (21.1%)
Galicia	1 (5.3%)
Basque Country	2 (10.5%)
Specialty	
Allergology	6 (31.6%)
Dermatology	3 (15.8%)
Immunology	2 (10.5%)
Pulmonology	4 (21.1%)
Otolaryngology	4 (21.1%)
Years of experience	
5–9 years	2 (10.5%)
10–19 years	4 (21.1%)
20–29 years	6 (31.6%)
Over 30 years	7 (36.8%)
Number of T2 patients monthly	
Fewer than 25 patients	1 (5.3%)
25–49 patients	5 (26.3%)
50–74 patients	3 (15.8%)
75–99 patients	1 (5.3%)
More than 100 patients	9 (47.4%)

The patient sample comprised 39 individuals who provided demographic and clinical data (Table 2). They were presented with the questionnaire version that had been validated by clinician consensus and featured the top-ranked language variants. Consensus was achieved on the ease of comprehension of the questionnaire items. The scientific committee examined the patients' comments from the first round and used them to make slight linguistic adjustments to specific terms. However, these changes were not deemed sufficiently significant to warrant another round of consultation, given that the comprehensibility of all items had already been agreed upon in the first round. This process led to the final validated version of the questionnaire (Supplementary Tables S1, S2). A summary of the results from the three rounds of the Delphi process is shown in Table 3.

**Table 2:** Demographic and clinical characteristics of the panel of patients.

Characteristics	Total (*N* = 39)
Age	
35–44 years	9 (23.1%)
45–54 years	9 (23.1%)
55–64 years	7 (17.9%)
Over 65 years	2 (5.1%)
Under 35 years	12 (30.8%)
Gender	
Male	11 (28.2%)
Female	28 (71.8%)
Region of residence	
Andalusia	5 (12.8%)
Aragon	1 (2.6%)
Canary Islands	1 (2.6%)
Castilla-La Mancha	1 (2.6%)
Castilla and León	1 (2.6%)
Catalonia	7 (17.9%)
Community of Madrid	7 (17.9%)
Navarre	1 (2.6%)
Valencian Community	4 (10.3%)
Extremadura	1 (2.6%)
Galicia	2 (5.1%)
Basque Country	8 (20.5%)
Which of these conditions do you have? If you have more than one, indicate the most severe	
Asthma	15 (38.5%)
Atopic Dermatitis	11 (28.2%)
NSAID–Exacerbated Respiratory Disease (N-ERD)	1 (2.6%)
Eosinophilic Esophagitis	9 (23.1%)
Chronic Rhinosinusitis with Nasal Polyps	3 (7.7%)
How many years ago were you diagnosed with this condition?	
10–19 years	9 (23.1%)
20–29 years	7 (17.9%)
5–9 years	7 (17.9%)
More than 30 years	7 (17.9%)
Less than 5 years	9 (23.1%)
Do you suffer from any other of these conditions? Mark here the one not selected in the previous question	
Food Allergy	8 (20.5%)
Asthma	9 (23.1%)
Atopic Dermatitis	5 (12.8%)
NSAID–Exacerbated Respiratory Disease (N-ERD)	2 (5.1%)
Eosinophilic Esophagitis	2 (5.1%)
Allergic Rhinitis	10 (25.6%)
Chronic Rhinosinusitis with Nasal Polyps	3 (7.7%)

**Table 3:** Summary of the results from the three rounds of the Delphi process.

Classification	*N*	%
Clinician's Delphi consultation: first round		
Appropriate	15	94%
Agreement	14	93%
Neutral	1	7%
Disagreement	0	0%
Uncertain	1	6%
Agreement	0	0%
Neutral	0	0%
Disagreement	1	100%
Inappropriate	0	0%
Agreement	0	
Neutral	0	
Disagreement	0	
Clinician's Delphi consultation: second round		
Appropriate	2	100%
Agreement	2	100%
Neutral	0	0%
Disagreement	0	0%
Uncertain	0	0%
Agreement	0	0%
Neutral	0	0%
Disagreement	0	0%
Inappropriate	0	0%
Agreement	0	0%
Neutral	0	0%
Disagreement	0	0%
Patient's Delphi consultation		
Appropriate	15	100%
Agreement	15	100%
Neutral	0	0%
Disagreement	0	0%
Uncertain	0	0%
Agreement	0	0%
Neutral	0	0%
Disagreement	0	0%
Inappropriate	0	0%
Agreement	0	0%
Neutral	0	0%
Disagreement	0	0%

## 4 Discussion

This study aimed to validate a previously developed screening tool to identify and facilitate early referral for T2 inflammation-related diseases (5), addressing critical gaps in interdisciplinary communication and early detection. Although the appropriate referral of patients has been primarily reported as a major challenge in the case of asthma (7), this issue seems equally relevant to other pathologies driven by T2 inflammation. In fact, in the specific case of patients with difficult-to-control asthma, identifying and effectively treating asthma comorbidities, including other T2-driven conditions such as chronic sinusitis with nasal polyps, may improve asthma control and reduce exacerbations (8). Furthermore, identifying comorbid T2 inflammatory conditions may help guide the optimal selection of biologic therapies (8).

The validation process results highlight the potential of this tool to prevent missing any coexisting T2 diseases and avoid delays in evaluation by the corresponding specialist, thereby improving patient care and outcomes. The Delphi technique is well-suited to the study's objective, as it has allowed many individuals from diverse locations and areas of expertise to participate anonymously, thus preventing the domination of the consensus process by one or a few experts (9). In this case, the role of the Delphi method is further enhanced by the collaborative strategy between clinicians and patients. In fact, including expert patients in the validation process is a crucial aspect of this study, as this approach has proven effective in other contexts (10, 11). The consensus achieved for most questionnaire items in the first Delphi round with clinicians in terms of comprehensibility and clinical relevance highlights the robustness of the questionnaire in identifying key indicators of T2 inflammatory diseases that warrant further evaluation.

Furthermore, affirming that a single positive response was sufficient to justify referral to a specialist is a significant advantage for this questionnaire. The only two items that initially failed to reach consensus were Question 1 (related to cough as a cardinal asthma symptom) and Question 13 (related to dysphagia in eosinophilic esophagitis). This was probably because the questions did not provide sufficient details and, as such, were unclear. However, after the scientific committee made consistent modifications regarding more specific signs, their duration, time of day or situation, and/or intensity, a consensus was achieved for these two items in the subsequent round. The ease of comprehension and the significance of patient input in refining the questionnaire after only one round, in which consensus was achieved for all items with a median score of 9, are remarkable. Moreover, the fact that the questionnaire's comprehensibility was evaluated by patients from different regions of Spain, each with distinct linguistic characteristics, further reinforces the validation results. Despite the relatively limited sample, the sociodemographic and clinical variables of the overall T2 population were adequately represented, except for patients over 65 years old who, as expected, were underrepresented.

Other authors have highlighted the need for improved diagnostic tools and multidisciplinary approaches to managing T2 inflammation-related diseases (12). A recent survey sponsored by the patient-driven T2i Network Project identified the common drivers and challenges related to the quality of life of patients with T2 inflammatory diseases from the patient's perspective (13). Senna *et al*. emphasized how a multidisciplinary team can serve as a central point for patient management, improving outcomes, reducing costs, and ensuring the most appropriate therapeutic decisions (14).

This validated questionnaire might therefore potentially help to overcome these unmet needs. It can be integrated into various healthcare settings, particularly primary care, although the questionnaire is designed to be adaptable across different healthcare levels, from primary care to specialized clinical settings, both in the public and private sectors. By providing a structured approach to symptom identification, this tool can enhance communication among specialists and primary care providers, ensuring that patients with suspected Type 2 inflammatory diseases are referred to the appropriate specialists without unnecessary delays. For example, in clinical practice, this questionnaire could be used as a screening tool in primary care consultations, facilitating early referral to specialized units. Moreover, in a hospital setting, its use by multidisciplinary teams would allow for systematic case identification and better coordination between different specialties, such as dermatology, ORL, or asthma units. This would ultimately lead to more personalized treatment strategies, particularly in selecting biological therapies tailored to individual patient profiles. A possible implementation model could involve its integration into the electronic medical record, with automated alerts for referrals when certain risk thresholds are met.

Despite its strengths, this study has limitations. The Delphi process itself may have introduced potential biases, for example, by including patient representatives from patient associations. These members may likely present with more severe forms of the disease and are usually more familiar with its clinical characteristics. This could skew the results, as the questionnaire is intended for early-stage diagnosis. However, it is worth noting that these patients may have severe forms of their primary diagnosed condition but not necessarily of the associated comorbidities. It is also worth considering whether the consensus decision to refer patients based on a single positive response could present a limitation. Even though this criterion simplifies decision-making, there is some uncertainty about whether it might lead to either unnecessary referrals or insufficient sensitivity to variations in disease severity, and further investigation is warranted. Another limitation concerns the unclear minimum age at which the questionnaire is valid. The current panel consisted of adult patients (over 18 years) who answered for themselves, and the questionnaire has not been validated with caregivers of pediatric patients. While it is reasonable to assume that caregivers will comprehend the items as adult patients did, specific symptoms may be more easily recognized in young children by caregivers, while others might be more challenging, especially in non-verbal pediatric patients. Nonetheless, the ability to implement the questionnaire in most patients would represent a significant advancement. Overall, these limitations should be considered when interpreting the findings, as they may affect their generalizability. The selection of experts and patients within the Delphi method may introduce biases, and the study's single-country scope, along with the exclusion of pediatric populations, may limit its applicability to broader, international, and younger patient groups. Therefore, although this method provides expert consensus on the development of the questionnaire, the external validity of our findings may be limited, as the composition of the panel may not fully represent the clinical variability in different healthcare settings.

Thus, several future actions are necessary to continue this line of research. First, further large-scale validation of this screening tool in larger, more diverse populations must confirm its applicability across different healthcare settings. Future studies should focus on prospective validation in real-world clinical practice, assessing its performance in various demographic and clinical contexts and its impact on referral accuracy and patient outcomes. Translating and validating the questionnaire across various languages and cultural contexts will be crucial for global use. Continuous refinement based on real-world application and feedback will further improve its accuracy and relevance. Additionally, ongoing education and clinician training will be essential to maximize the questionnaire's effectiveness. Finally, integrating the tool into electronic health records could greatly enhance accessibility and ease of use, paving the way for its broader implementation in clinical practice.

## 5 Conclusions

This screening questionnaire provides a simple and practical tool for the early detection of coexisting T2 inflammatory diseases, enhancing comprehensive and personalized patient care by increasing multidisciplinary efforts and accelerating diagnosis. The next phase will involve evaluating the questionnaire's effectiveness in real-world clinical settings to determine its practical utility.

## Data Availability

The original contributions presented in the study are included in the article/Supplementary Material, further inquiries can be directed to the corresponding author.
