# Supplementary material for: Development and validation of a Delphi consensus-based questionnaire for the multidisciplinary management of type 2 inflammation-related diseases
Source: Front Allergy. 2025 Apr 3;6:1543504. doi: 10.3389/falgy.2025.1543504 (PMC12003428; doi:10.3389/falgy.2025.1543504)
Supplement: Supplementary file 1 [file Table1.docx]

**Table S1.** Final questionnaire validated for use in Spain for initial screening of T2 inflammation-associated pathologies (Spanish version)

| **Pregunta ^a^** | **Respuesta (sí/no)** | **Sospecha diagnóstica ^b, c^** |
| --- | --- | --- |
| 1. ¿Tiene tos seca (sin flemas) de manera continua o durante más de un mes, sobre todo nocturna? |  | Asma |
| 1. ¿Le falta el aire incluso sin haber hecho esfuerzo físico? |  |  |
| 1. ¿Le cuesta respirar por la nariz de manera habitual? |  | Rinosinusitis crónica con pólipos nasales |
| 1. ¿Nota que ha perdido olfato o huele menos las cosas? |  |  |
| 1. ¿Estornuda muchas veces seguidas de forma habitual o durante algunos periodos del año? |  | Rinitis alérgica |
| 1. ¿Le pica la nariz y/o el paladar de forma habitual o durante algunos periodos del año? |  |  |
| 1. ¿Le pican los ojos de forma habitual o durante algunos periodos del año? |  | Conjuntivitis alérgica |
| 1. ¿Nota los ojos enrojecidos y/o llorosos de forma habitual o durante algunos periodos del año? |  |  |
| 1. Después de comer algún alimento, ¿ha notado picor o comezón en los labios, la boca, o la garganta? |  | Alergia alimentaria mediada por IgE |
| 1. Después de comer algún alimento, ¿ha sentido de forma repentina dificultad para respirar, mareo, picor por el cuerpo, hinchazón en la cara, o vómitos? |  |  |
| 1. ¿Tiene sarpullidos o manchas rojas (eczema o dermatitis) en la piel que le producen mucho picor con frecuencia o durante mucho tiempo del año? |  | Dermatitis atópica |
| 1. ¿Nota la piel con descamación o seca en las zonas donde se rasca frecuentemente? |  |  |
| 1. ¿En ocasiones tiene problema para tragar los alimentos o incluso presenta náuseas y vómitos? |  | Esofagitis eosinofílica |
| 1. Al ingerir alimento, ¿en ocasiones nota una especie de nudo o molestia en el pecho/boca del estómago que no le permite seguir tragando? |  |  |
| 1. ¿Ha sentido empeoramiento de síntomas respiratorios (congestión, sensación de ahogo, agüilla en la nariz…) al poco tiempo (1-3 horas) de tomarse una aspirina, ibuprofeno u otro antiinflamatorio? |  | EREA |

^a^ Las preguntas están formuladas con un lenguaje adaptado a la fácil comprensión de cualquier persona o paciente sin formación médica

^b^ La derivación debería considerarse en caso de al menos una respuesta afirmativa

^c^ La versión para pacientes no incluirá la columna de sospecha diagnóstica

EREA, enfermedad respiratoria exacerbada por AINE

**Table S2.** Final questionnaire validated for its use in Spain for initial screening of T2 inflammation-associated pathologies (translation to English*)

| **Question ^a^** | **Answer (yes/no)** | **Suspected diagnosis ^b, c^** |
| --- | --- | --- |
| 1. Do you have a dry cough (without phlegm) continuously or for more than a month, especially at night? |  | Asthma |
| 1. Do you feel short of breath even without physical exertion? |  |  |
| 1. Do you habitually have difficulty breathing through your nose? |  | Chronic rhinosinusitis with nasal polyps |
| 1. Have you noticed a loss of smell or a decreased ability to smell things? |  |  |
| 1. Do you frequently sneeze many times in a row or during certain periods of the year? |  | Allergic rhinitis |
| 1. Do you frequently experience itching in your nose and/or palate or during certain periods of the year? |  |  |
| 1. Do your eyes frequently itch or during certain periods of the year? |  | Allergic conjunctivitis |
| 1. Do your eyes appear red and/or watery frequently or during specific periods of the year? |  |  |
| 1. After eating a particular food, have you noticed itching or tingling in your lips, mouth, or throat? |  | IgE-mediated food allergy |
| 1. After eating a particular food, have you suddenly experienced difficulty breathing, dizziness, body itching, facial swelling, or vomiting? |  |  |
| 1. Do you frequently have rashes or red patches (eczema or dermatitis) on your skin that itch a lot or for long periods of the year? |  | Atopic dermatitis |
| 1. Do you notice flaky or dry skin in areas where you frequently scratch? |  |  |
| 1. Do you sometimes have trouble swallowing food or even experience nausea and vomiting? |  | Eosinophilic esophagitis |
| 1. When eating, do you sometimes feel a lump or discomfort in your chest/stomach that prevents you from continuing to swallow? |  |  |
| 1. Have you experienced worsening respiratory symptoms (congestion, shortness of breath, runny nose) shortly (1-3 hours) after taking aspirin, ibuprofen, or another anti-inflammatory? |  | NERD |

* This English version is merely a translation of the validated Spanish questionnaire. It has not yet been validated for use in countries where English is the primary language; this would be a future approach to consider.

^a^ The questions are formulated in a language easily understandable by any person or patient without medical training.

^b^ Referral should be considered if at least one question is answered affirmatively.

^c^ The patient version will not include the suspected diagnosis column.

NERD, nonsteroidal anti-inflammatory drug-exacerbated respiratory disease

**Table S3a.** Detailed numerical results of the ranking of alternative statements submitted for consultation in round 1 of the clinicians' panel (Spanish version used during the Delphi process).

| **Pregunta** | **Posición** | **Opción** | **N** | **1ª** | **2ª** | **3ª** | **4ª** | **5ª** | **Puntos** |
| --- | --- | --- | --- | --- | --- | --- | --- | --- | --- |
| 1 | 1 | b. ¿Tiene tos seca (sin flemas) de manera continua o durante más de un mes, sobre todo nocturna? | 19 | 14 | 3 | 2 | 0 | 0 | 88 |
| 1 | 2 | a. ¿Tienes tos seca de manera contínua o durante más de un mes? | 19 | 3 | 8 | 4 | 1 | 3 | 64 |
| 1 | 3 | d. ¿Tiene tos sin expectoración de manera continua o durante más de un mes? | 19 | 1 | 4 | 3 | 8 | 3 | 49 |
| 1 | 4 | e. ¿Tiene tos irritativa de manera continua o durante más de un mes? | 19 | 1 | 2 | 5 | 5 | 6 | 44 |
| 1 | 5 | c. ¿Tiene tos no productiva de manera continua o durante más de un mes? | 19 | 0 | 2 | 5 | 5 | 7 | 40 |
| 2 | 1 | b. ¿Le falta el aire incluso sin haber hecho esfuerzo físico? | 16 | 10 | 2 | 4 |  |  | 38 |
| 2 | 2 | a. ¿Le cuesta respirar incluso sin haber hecho esfuerzo físico? | 16 | 3 | 10 | 3 |  |  | 32 |
| 2 | 3 | c. ¿No le entra bien todo el aire incluso sin haber hecho esfuerzo físico? | 16 | 3 | 4 | 9 |  |  | 26 |
| 3 | 1 | c. ¿Le cuesta respirar por la nariz de manera habitual? | 17 | 8 | 7 | 2 |  |  | 40 |
| 3 | 2 | a. ¿Tiene la nariz congestionada de forma habitual? | 17 | 5 | 4 | 8 |  |  | 31 |
| 3 | 3 | b. ¿Tiene la nariz obstruida de forma habitual? | 17 | 4 | 6 | 7 |  |  | 31 |
| 4 | 1 | b. ¿Nota que ha perdido olfato o huele menos las cosas? | 13 | 10 | 1 | 2 |  |  | 34 |
| 4 | 2 | a. ¿Siente que ha perdido el olfato o huele peor las cosas? | 13 | 2 | 10 | 1 |  |  | 27 |
| 4 | 3 | c. ¿Siente que ha empeorado su olfato de forma relevante? | 13 | 1 | 2 | 10 |  |  | 17 |
| 5 | 1 | b. ¿Estornuda muchas veces seguidas de forma habitual o durante algunos periodos del año? | 16 | 16 | 0 |  |  |  | 32 |
| 5 | 2 | a. ¿Estornuda con mucha frecuencia de forma habitual o durante algunos periodos del año? | 16 | 0 | 16 |  |  |  | 16 |
| 6 | 1 | a.  ¿Le pica la nariz y/o el paladar de forma habitual o durante algunos periodos del año? | 19 | 10 | 9 |  |  |  | 29 |
| 6 | 2 | b. ¿Nota picores en el paladar y/o en la nariz de forma habitual o durante algunos periodos de tiempo? | 19 | 9 | 10 |  |  |  | 28 |
| 7 | 1 | b. Después de comer algún alimento, ¿ha notado picor o comezón en los labios, la boca, o la garganta? | 9 | 9 | 0 |  |  |  | 18 |
| 7 | 2 | a. Después de comer algún alimento, ¿ha notado picor en los labios, la boca, o la garganta? | 9 | 0 | 9 |  |  |  | 9 |
| 8 | 1 | a. Después de comer algún alimento, ¿ha sentido de forma repentina dificultad para respirar, mareo, picor por el cuerpo, hinchazón en la cara, o vómitos? | 17 | 6 | 3 | 6 | 2 |  | 47 |
| 8 | 2 | d. Después de comer algún alimento, ¿ha sentido de forma repentina dificultad para respirar, mareo, picor, hinchazón en la cara, o lesiones en la piel? | 17 | 5 | 3 | 5 | 4 |  | 43 |
| 8 | 3 | c. Después de comer algún alimento, ¿ha sentido de forma repentina sensación de ahogo, mareo, picor por el cuerpo, hinchazón en la cara, o vómitos? | 17 | 4 | 5 | 2 | 6 |  | 41 |
| 8 | 4 | b. Después de comer algún alimento, ¿ha sentido de forma repentina dificultad para respirar, mareo, picor o comezón por el cuerpo, hinchazón en la cara, o vómitos? | 17 | 2 | 6 | 4 | 5 |  | 39 |
| 9 | 1 | a. ¿Tiene sarpullidos o manchas rojas (eczema o dermatitis) en la piel que le producen mucho picor con frecuencia o durante mucho tiempo del año? | 9 | 6 | 3 |  |  |  | 15 |
| 9 | 2 | b. ¿Tiene sarpullidos o manchas rojas en la piel que le producen mucho picor o comezón con frecuencia? | 9 | 3 | 6 |  |  |  | 12 |
| 10 | 1 | b. ¿Nota la piel con descamación o seca en las zonas donde se rasca frecuentemente? | 19 | 13 | 6 |  |  |  | 32 |
| 10 | 2 | a. ¿Nota la piel más gruesa o rasposa en las zonas donde se rasca frecuentemente? | 19 | 6 | 13 |  |  |  | 25 |
| 11 | 1 | b. ¿En ocasiones tiene problema para tragar los alimentos o incluso presenta náuseas y vómitos? | 12 | 11 | 1 |  |  |  | 23 |
| 11 | 2 | a. ¿En ocasiones tiene problema para tragar los alimentos o incluso presenta náuseas y regurgitaciones? | 12 | 1 | 11 |  |  |  | 13 |
| 12 | 1 | a. ¿Ha sentido empeoramiento de síntomas respiratorios (congestión, dificultad para respirar, goteo por la nariz…) al poco tiempo (1-3 horas) de tomarse una aspirina, ibuprofeno u otro antiinflamatorio? | 15 | 7 | 5 | 3 |  |  | 34 |
| 12 | 2 | b. ¿Ha sentido empeoramiento de síntomas respiratorios (congestión, sensación de ahogo, goteo por la nariz…) al poco tiempo (1-3 horas) de tomarse una aspirina, ibuprofeno u otro analgésico (pastilla para los dolores)? | 15 | 7 | 4 | 4 |  |  | 33 |
| 12 | 3 | c. ¿Ha sentido empeoramiento de síntomas respiratorios (congestión, sensación de ahogo, agüilla en la nariz…) al poco tiempo (1-3 horas) de tomarse una aspirina, ibuprofeno u otro antiinflamatorio? | 15 | 1 | 6 | 8 |  |  | 23 |

**Table S3b.** Detailed numerical results of the ranking of alternative statements submitted for consultation in round 1 of the clinicians' panel (translation to English).

| **Question** | **Position** | **Option** | **N** | **1st** | **2nd** | **3rd** | **4th** | **5th** | **Points** |
| --- | --- | --- | --- | --- | --- | --- | --- | --- | --- |
| 1 | 1 | b. Do you have a dry cough (without phlegm) continuously or for more than a month, especially at night? | 19 | 14 | 3 | 2 | 0 | 0 | 88 |
| 1 | 2 | a. Do you have a dry cough continuously or for more than a month? | 19 | 3 | 8 | 4 | 1 | 3 | 64 |
| 1 | 3 | d. Do you have a cough without expectoration continuously or for more than a month? | 19 | 1 | 4 | 3 | 8 | 3 | 49 |
| 1 | 4 | e. Do you have an irritating cough continuously or for more than a month? | 19 | 1 | 2 | 5 | 5 | 6 | 44 |
| 1 | 5 | c. Do you have a non-productive cough continuously or for more than a month? | 19 | 0 | 2 | 5 | 5 | 7 | 40 |
| 2 | 1 | b. Do you experience shortness of breath even without physical exertion? | 16 | 10 | 2 | 4 |  | 0 | 38 |
| 2 | 2 | a. Do you find it hard to breathe even without physical exertion? | 16 | 3 | 10 | 3 |  | 0 | 32 |
| 2 | 3 | c. Do you have difficulty getting enough air even without physical exertion? | 16 | 3 | 4 | 9 |  | 0 | 26 |
| 3 | 1 | c. Do you have difficulty breathing through your nose habitually? | 17 | 8 | 7 | 2 |  | 0 | 40 |
| 3 | 2 | a. Is your nose habitually congested? | 17 | 5 | 4 | 8 |  | 0 | 31 |
| 3 | 3 | b. Is your nose habitually blocked? | 17 | 4 | 6 | 7 |  | 0 | 31 |
| 4 | 1 | b. Have you noticed a loss of smell or reduced ability to smell things? | 13 | 10 | 1 | 2 |  | 0 | 34 |
| 4 | 2 | a. Do you feel that you have lost your sense of smell or that things smell worse? | 13 | 2 | 10 | 1 |  | 0 | 27 |
| 4 | 3 | c. Do you feel that your sense of smell has significantly worsened? | 13 | 1 | 2 | 10 |  | 0 | 17 |
| 5 | 1 | b. Do you sneeze many times in a row habitually or during certain periods of the year? | 16 | 16 | 0 | 0 |  | 0 | 32 |
| 5 | 2 | a. Do you sneeze very frequently habitually or during certain periods of the year? | 16 | 0 | 16 | 0 |  | 0 | 16 |
| 6 | 1 | a. Do you have itching in your nose and/or palate habitually or during certain periods of the year? | 19 | 10 | 9 | 0 |  | 0 | 29 |
| 6 | 2 | b. Do you experience itching in your palate and/or nose habitually or during certain periods of time? | 19 | 9 | 10 | 0 |  | 0 | 28 |
| 7 | 1 | b. After eating any food, have you noticed itching or a rash on your lips, mouth, or throat? | 9 | 9 | 0 | 0 |  | 0 | 18 |
| 7 | 2 | a. After eating any food, have you noticed itching on your lips, mouth, or throat? | 9 | 0 | 9 | 0 |  | 0 | 9 |
| 8 | 1 | a. After eating any food, have you suddenly felt difficulty breathing, dizziness, itching all over your body, facial swelling, or vomiting? | 17 | 6 | 3 | 6 | 2 | 0 | 47 |
| 8 | 2 | d. After eating any food, have you suddenly felt difficulty breathing, dizziness, itching, facial swelling, or skin lesions? | 17 | 5 | 3 | 5 | 4 | 0 | 43 |
| 8 | 3 | c. After eating any food, have you suddenly felt a sensation of choking, dizziness, itching all over your body, facial swelling, or vomiting? | 17 | 4 | 5 | 2 | 6 | 0 | 41 |
| 8 | 4 | b. After eating any food, have you suddenly felt difficulty breathing, dizziness, itching or rash all over your body, facial swelling, or vomiting? | 17 | 2 | 6 | 4 | 5 | 0 | 39 |
| 9 | 1 | a. Do you have rashes or red spots (eczema or dermatitis) on your skin that cause frequent or prolonged itching throughout the year? | 9 | 6 | 3 | 0 |  | 0 | 15 |
| 9 | 2 | b. Do you have rashes or red spots on your skin that cause frequent itching? | 9 | 3 | 6 | 0 |  | 0 | 12 |
| 10 | 1 | b. Do you notice peeling or dry skin in areas where you frequently scratch? | 19 | 13 | 6 | 0 |  | 0 | 32 |
| 10 | 2 | a. Do you notice thicker or rougher skin in areas where you frequently scratch? | 19 | 6 | 13 | 0 |  | 0 | 25 |
| 11 | 1 | b. Do you sometimes have trouble swallowing food or even experience nausea and vomiting? | 12 | 11 | 1 | 0 |  | 0 | 23 |
| 11 | 2 | a. Do you sometimes have trouble swallowing food or even experience nausea and regurgitation? | 12 | 1 | 11 | 0 |  | 0 | 13 |
| 12 | 1 | a. Have you experienced worsening respiratory symptoms (congestion, difficulty breathing, nasal drip, etc.) shortly after taking aspirin, ibuprofen, or another anti-inflammatory? | 15 | 7 | 5 | 3 |  | 0 | 34 |
| 12 | 2 | b. Have you experienced worsening respiratory symptoms (congestion, sensation of choking, nasal drip, etc.) shortly after taking aspirin, ibuprofen, or another pain reliever (pain pill)? | 15 | 7 | 4 | 4 |  | 0 | 33 |
| 12 | 3 | c. Have you experienced worsening respiratory symptoms (congestion, sensation of choking, nasal drip, etc.) shortly after taking aspirin, ibuprofen, or another anti-inflammatory? | 15 | 1 | 6 | 8 |  | 0 | 23 |
